# Supplementary material for: Teaching methods for critical thinking in health education of children up to high school: A scoping review
Source: PLoS One. 2024 Jul 18;19(7):e0307094. doi: 10.1371/journal.pone.0307094 (PMC11257347; doi:10.1371/journal.pone.0307094)
Supplement: S2 Table — (DOCX) [file pone.0307094.s002.docx]

**Supporting information 2**

**Search strategies.**

| **SEARCH STRATEGY Ovid MEDLINE(R)** and Epub Ahead of Print, In-Process, In-Data-Review & Other Non-Indexed Citations, Daily and Versions(R) |
| --- |
| 1 exp Health Knowledge, Attitudes, Practice/ or exp Health Literacy/ or exp Health Education/  2 critical thinking.mp. or exp Thinking/  3 Thought.mp.  4 thinking skill.mp.  5 (thinking skill or thinking skills).mp.  6 *Schools/ed [Education]  7 school*.mp. or *Schools/  8 primary school.mp.  9 secondary school.mp.  10 elementary school.mp.  11 high school.mp.  12 middle school.mp.  13 exp Judgment/ or judgement.mp.  14 metacognition.mp. or exp Metacognition/  15 (Metaemotion or Metamemory or Metamemories or Metacognitive Knowledge or Metacognitive Awarenesses).mp.  16 Choice Behavior.mp. or *Choice Behavior/ or informed choice.mp. or informed choices.mp.  17 Approach Behavior.mp.  18 *Decision Making/ or Decision Making.mp.  19 2 or 3 or 4 or 5 or 13 or 14 or 15 or 16 or 17 or 18  20 6 or 7 or 8 or 9 or 10 or 11 or 12  21 curriculum.mp. or *Curriculum/  22 school curriculum.mp.  23 *Teaching/ed, mt, og, px, st, td [Education, Methods, Organization & Administration, Psychology, Standards, Trends]  24 (educational technique or educational techniques).mp.  25 (Educational Technic or Educational Technics).mp.  26 (Teaching Method or Teaching Methods).mp.  27 Pedagogy.mp.  28 Pedagogies.mp.  29 (Training Activity or Training Activities).mp.  30 21 or 22 or 23 or 24 or 25 or 26 or 27 or 28 or 29  31 20 and 30  32 1 and 19 and 31 |
| **SEARCH STRATEGY WEB OF SCIENCE FROM CLARIVATE** |
| AK=("health literacy") OR AK=(health near/3 education) OR AK=("health behavior") OR TI=("health literacy") OR TI=(health near/3 education) OR TI=("health behavior") OR AB=("health literacy") OR AB=(health near/3 education) OR AB=("health behavior")  AK=("critical thinking") OR AK=("decision making") OR AK=("informed choice") OR TI=("critical thinking") OR TI=("decision making") OR TI=("informed choice") OR AB=("critical thinking") OR AB=("decision making") OR AB=("informed choice")  AK=(school) OR AK=(teaching) OR AK=(education) OR TI=(school) OR TI=(teaching) OR TI=(education) OR AB=(school) OR AB=(teaching) OR AB=(education)  AK=(curriculum) OR AK=("teaching methods") OR AK=(education) OR AK=(pedagogy) OR AK=("teaching program") |
| **SEARCH STRATEGY ERIC FROM EBSCO** |
| (DE "Health Promotion") AND (DE "Comprehensive School Health Education") OR (DE "Health Literacy") AND (DE "Curriculum") OR (DE "Critical Thinking" AND DE "Teaching Methods") |
| **SEARCH STRATEGY CINAHL FROM EBSCO** |
| ((MM "Critical Thinking")OR "critical thinking" OR(MM "Decision Making")OR (MH "Thinking+")OR (MH "ProblemSolving+") OR (MM "Judgment") OR (MM"Problem Identification")OR (MH"Brainstorming") OR OR (MH "HealthEducation+") OR (MM"School HealthEducation") OR (MM"Student HealthEducation") OR (MH"Health Information+")OR (MH "InformationLiteracy+") OR (MM"Health Literacy")) AND ((MH "Schools,Elementary") OR (MH"Schools, Middle") OR(MH "Schools,Secondary") OR (MH"Students, Elementary")OR (MH "Students,Middle School") OR (MH"Students, High School") OR (MH "Education,Nonprofessional")) AND ((MH "TeachingMaterials+") OR (MH"Teaching+") OR (MH "LearningMethods+") OR (MH"Curriculum+") OR (MH"Teaching+") OR (MH "TeachingMethods+") OR (MH"Teaching+")) |
| **SEARCH STRATEGY EMBASE FROM ELSEVIER** |
| #16,"('critical thinking' OR 'decision making' OR 'health behavior' OR 'skill' OR 'metacognition') AND ('health literacy'/exp/mj OR 'health education'/exp/mj) AND (('health literacy'/exp/mj OR 'health education'/exp/mj) AND ([adolescent]/lim OR [school]/lim)) AND ('teaching' OR 'curriculum' OR 'education')"  #15,"(('health literacy'/exp/mj OR 'health education'/exp/mj) AND ([adolescent]/lim OR [school]/lim)) AND ('critical thinking' OR 'thinking') AND ('teaching' OR 'curriculum' OR 'education')"  #14,"('health literacy'/exp/mj OR 'health education'/exp/mj) AND (('health literacy'/exp/mj OR 'health education'/exp/mj) AND ([adolescent]/lim OR [school]/lim)) AND ('teaching' OR 'curriculum' OR 'education')"  #13,"('health literacy'/exp/mj OR 'health education'/exp/mj) AND (('health literacy'/exp/mj OR 'health education'/exp/mj) AND ([adolescent]/lim OR [school]/lim)) AND ('critical thinking' OR 'thinking') AND ('teaching' OR 'curriculum' OR 'education')"  #12,"'teaching' OR 'curriculum' OR 'education'"  #11,"'critical thinking' OR 'thinking'"  #10,"('health literacy'/exp/mj OR 'health education'/exp/mj) AND ([adolescent]/lim OR [school]/lim)"  #9,"'health literacy'/exp/mj OR 'health education'/exp/mj"  #8,"(('health'/de AND 'education'/de OR 'health'/de) AND 'literacy'/de OR 'health promotion'/de) AND ((('health'/de AND 'education'/de OR 'health'/de) AND 'literacy'/de OR 'health promotion'/de) AND ([adolescent]/lim OR [school]/lim)) AND ('critical thinking' OR 'decision making' OR 'health behavior' OR 'skill' OR 'metacognition') AND ('teaching' OR 'curriculum' OR 'education program')"  #7,"(('health'/de AND 'education'/de OR 'health'/de) AND 'literacy'/de OR 'health promotion'/de) AND ((('health'/de AND 'education'/de OR 'health'/de) AND 'literacy'/de OR 'health promotion'/de) AND ([adolescent]/lim OR [school]/lim)) AND ('teaching' OR 'curriculum' OR 'education program')"  #6,"(('health'/de AND 'education'/de OR 'health'/de) AND 'literacy'/de OR 'health promotion'/de) AND ('critical thinking' OR 'decision making' OR 'health behavior' OR 'skill' OR 'metacognition') AND ('teaching' OR 'curriculum' OR 'education program')"  #5,"(('health'/de AND 'education'/de OR 'health'/de) AND 'literacy'/de OR 'health promotion'/de) AND ('teaching' OR 'curriculum' OR 'education program')"  #4,"'teaching' OR 'curriculum' OR 'education program'"  #3,"'critical thinking' OR 'decision making' OR 'health behavior' OR 'skill' OR 'metacognition'"  #2,"(('health'/de AND 'education'/de OR 'health'/de) AND 'literacy'/de OR 'health promotion'/de) AND ([adolescent]/lim OR [school]/lim)"  #1,"('health'/de AND 'education'/de OR 'health'/de) AND 'literacy'/de OR 'health promotion'/de" |
| **SEARCH STRATEGY PROQUEST FROM PROQUEST CENTRAL** |

| Set# | Searched for | Databases |
| --- | --- | --- |
| S1 | MAINSUBJECT.EXACT("Health education") OR MAINSUBJECT.EXACT("Health behavior") OR MAINSUBJECT.EXACT("Health literacy") OR mainsubject(health AND information) OR mainsubject(health claims) AND mainsubject(health claim) | Coronavirus Research Database, ProQuest Central, ProQuest Dissertations & Theses A&I, Publicly Available Content Database |
| S2 | MAINSUBJECT.EXACT("Critical thinking") OR MAINSUBJECT.EXACT("Skills") OR (MAINSUBJECT.EXACT("Judgments") OR MAINSUBJECT.EXACT("Judgment")) OR MAINSUBJECT.EXACT("Metacognition") OR MAINSUBJECT.EXACT("Health behavior") OR MAINSUBJECT.EXACT("Decision making") OR MAINSUBJECT.EXACT("Cognitive ability") OR MAINSUBJECT.EXACT("Cognition & reasoning") | Coronavirus Research Database, ProQuest Central, ProQuest Dissertations & Theses A&I, Publicly Available Content Database |
| S3 | (MAINSUBJECT.EXACT("Secondary schools") OR MAINSUBJECT.EXACT("Education")) OR (MAINSUBJECT.EXACT("Elementary schools") OR MAINSUBJECT.EXACT("Schools")) OR (MAINSUBJECT.EXACT("Middle school education") OR MAINSUBJECT.EXACT("Middle schools")) OR MAINSUBJECT.EXACT("Elementary schools") OR (MAINSUBJECT.EXACT("Students") OR MAINSUBJECT.EXACT("Secondary school students") OR MAINSUBJECT.EXACT("Elementary school students")) | Coronavirus Research Database, ProQuest Central, ProQuest Dissertations & Theses A&I, Publicly Available Content Database |
| S4 | MAINSUBJECT.EXACT("Core curriculum") OR (MAINSUBJECT.EXACT("Teaching methods") OR MAINSUBJECT.EXACT("Education") OR MAINSUBJECT.EXACT("Student teaching") OR MAINSUBJECT.EXACT("Pedagogy")) OR MAINSUBJECT.EXACT("Teaching") | Coronavirus Research Database, ProQuest Central, ProQuest Dissertations & Theses A&I, Publicly Available Content Database |
| S5 | S3 AND S4 | Coronavirus Research Database, ProQuest Central, ProQuest Dissertations & Theses A&I, Publicly Available Content Database  These databases are searched for part of your query. |
| S6 | S1 AND S2 AND S5 | Coronavirus Research Database, ProQuest Central, ProQuest Dissertations & Theses A&I, Publicly Available Content Database  These databases are searched for part of your query. |
