# Supplementary material for: Teaching methods for critical thinking in health education of children up to high school: A scoping review
Source: PLoS One. 2024 Jul 18;19(7):e0307094. doi: 10.1371/journal.pone.0307094 (PMC11257347; doi:10.1371/journal.pone.0307094)
Supplement: S3 Table — (DOCX) [file pone.0307094.s003.docx]

**Supporting information 3**

| **Characteristics of the included studies.** | | | | | | | | | |
| --- | --- | --- | --- | --- | --- | --- | --- | --- | --- |
|  | **Study ID** | **Years of conducting the study** | **Country** | **Health issue** | **Intervention populations** | **Pupils’ age** in years or **grade of education** | **Gender**  **(% Female)** | **Type of study** | **Research design, method/ technique** |
| **1** | **Aghazadeh 2020 [111]** | NR | USA | Public health, physical activity, nutrition | 5 schools, 365 pupils, 5 teachers | 8 years | 44.4 | Mixed methods | Single arm before and after, individual semi-structed interview |
| **2** | **Anderson 2005 [22]** | 1999-2000 | Scotland | Nutrition | 4 schools, 511 pupils | 6-7, 10-11 years | 54 | Quantitative | Cluster randomized |
| **3** | **Alekseeva 2015 [112]** | 2009-2011 | Russia | SRH: AIDS/HIV | 969 schools, 98000 pupils, | 13-23 years | NR | Mixed methods | Cross-sectional survey, individual semi-structured interview |
| **4** | **Allsop 2022 [74]** | NR | USA | SRH | 4 schools, 561 pupils | 9-12 grades | 43.5 | Quantitative | Cross-sectional survey |
| **5** | **Arauz Ledezma 2021[142]** | NR | Panama | Mental health | 1 school, 48 pupils, 3 teachers | 12-15 years | 55 | Qualitative | Interview/focus group |
| **6** | **Araujo 2017 [23]** | 2011-2014 | Portugal | Public health | 15 schools,  2215 pupils, 215 teachers | NR | NR | Quantitative | Cross-sectional survey |
| **7** | **Audrey 2006 [90]** | 2001-2004 | UK | Psychoactive substance use: tobacco | 30 schools, 5358 pupils | 12-13 years | 50.7 | Mixed methods | Cluster randomized and Interview/ focus group/ |
| **8** | **Aventin 2020 [91]** | 2018-2019 | UK | SRH | 8 schools, 134 parents | 14-16 years | 52 | Mixed methods | Cluster randomized, survey and individual semi-structured interviews, focus groups |
| **9** | **Banas 2021[114]** | 2019 | USA | Mental health | 133 pupils | 10^th^ grade | NR | Mixed  methods | Cross-sectional survey and journal entries |
| **10** | **Basen-Engquis 1997 [69], Coyle 1999 [144]** | 1993-1995 | USA | SRH: AIDS/HIV | 20 schools, 3869 pupils | 9-10 grades | 53 | Quantitative | Cluster randomized |
| **11** | **Bell R 1993 [136]** | 1984-1986 | USA | Psychoactive substance use: drugs | 30 schools, 6527 pupils | 7–9 grades | 49 | Quantitative | Quasi-experimental |
| **12** | **Bell M 2005 [68]** | 1999/2000-2002/2003 | USA | Psychoactive substance use: alcohol | 8 schools, 1214 pupils | 4-5 grades | 51 | Quantitative | Quasi-experimental |
| **13** | **Begoray 2009 [135]** | 2005-2006 | Canada | Somatic health | 4 schools, 33 pupils | 14-15 Years | 55 | Qualitative | Interview/focus group/group interview |
| **14** | **Bond 2004 [57]** | 1997-1999 | Australia | Psychoactive substance use: drugs | 26 schools, 2678 pupils | 13-16 years | 52.25 | Quantitative | Cluster randomized |
| **15** | **Bonnesen 2023** | 2016-2017 | Denmark | Mental health | 14 schools, 1388 pupils | 16 years | 63,9 | Quantitative | Cluster randomized |
| **16** | **Borawski 2009 [58]** | 2000-2002 | USA | SRH: AIDS/HIV | 10 schools, 1576 pupils | 15 years | 51.8 | Quantitative | Cluster randomized |
| **17** | **Brinez 2019 [130]** | 2015 | Columbia | SRH | 1 school, 66 pupils, 1 teacher | 12-16 years | NA | Qualitative | Action research |
| **18** | **Brotman 2013 [129]** | NR | USA | SRH | 1 school, NR | 16-18 years | 90 | Qualitative | Ethnographic research methods, Interview, focus group, participant observation interview |
| **19** | **Bruselius-Jensen 2014, 2017 [117, 119]** | 2013-2014 | Denmark | Physical activity | 4 schools, 281 pupils, 9 teachers | 10-13 years | NR | Qualitative | Discourse analysis |
| **20** | **Bruselius-Jensen 2017 [118]** | 2014-2015 | Denmark | Public health | 4 schools  108 pupils | 10-13 years | NR | Qualitative | Interview/ focus group/group interview |
| **21** | **Byers 2003 [70]** | 2001 | Canada | SRH | 10 schools, 745 pupils | 6-8 grades | 54 | Quantitative | Cross-sectional survey |
| **22** | **Caria 2011 [24]** | 2004-2006 | Austria | Psychoactive substance use: Alcohol | 143 schools  5541 pupils | 12-14 years | 49 | Quantitative | Cluster randomized, survey |
| **23** | **Carlsson 2012 [145]** | 2006-2008 | Netherlands, Spain, Italy, Denmark, Austria | Nutrition, physical activity | 73 schools, 2500-2700 pupils | 11-16 years | NR | Qualitative | Document analysis, observation, interviews, group interviews |
| **24** | **Carolan 2007 [71]** | 2001 | USA | Psychoactive substance use: tobacco | NR | 6th grade | 52 | Quantitative | Individually randomized |
| **25** | **Cheng 2008 [92]** | 2003 | China | SRH: AIDS/HIV | 2 schools, 717 pupils, 28 teachers | 14-18 years | 42 | Mixed methods | Quasi experimental and interview, focus group, group interview |
| **26** | **Contento 2007 [72]** | NR | USA | Nutrition, physical activity | 5 schools, 278 pupils | 11-13 years | NR | Quantitative | Uncontrolled before-and-after |
| **27** | **Cooper 2022 [77]** | 2019- 2020 | UK | Public health | 10 schools, 1162 pupils | NR | NR | Quantitative | Stepped-wedged design |
| **28** | **Davies 2023 [78]** | 2013-2015 | Australia | Public health | 21 schools, 6967 pupils | 12-13 years | NR | Quantitative | Cluster-randomized trial |
| **29** | **Dela Fuente-Anuncibay 2023 [79]** | 2015-2016 | Italy | Nutrition | 14 schools,  185 pupils  55 teachers | 7-10 years | 48 | Quantitative | Quasi experimental |
| **30** | **Denny 2006 [25]** | NR | USA | SRH | 830 pupils | 5- 9 and above grades | NR | Quantitative | Quasi-experimental |
| **31** | **DiCicco 1984 [93]** | 1975-1979 | USA | Psychoactive substance use: alcohol | ~1539 pupils (matched control group design: 490, 25alternate approach: 386; long term effects: 571; cross-sectional: 92 pupils) | 7, 8, 10, 12 grades | NR | Mixed methods | Controlled-before and after and participant observation, interviews |
| **32** | **Dinaj-Koci 2015 [35]** | 2004-2005 and 2008- 2009 | The Bahamas | SRH: AIDS/HIV | 15 schools, 537 pupils | 6th grade | 56.6 | Quantitative | Individually randomized |
|  |  |  |  |  | 8 schools, 2593 pupils | 10th grade | 52.7 |  |  |
| **33** | **Dunton 2012 [36]** | 2010–2011 | USA | Nutrition | 22 schools, 195245 pupils, 7359 teachers | 8 years | 52 | Quantitative | Controlled-before and after |
| **34** | **Fage-Butler 2019 [123]** | NR | Scotland | Public health: violence | NR | 10-12 years | NR | Qualitative | Action research |
| **35** | **Flay 1985 [37]** | 1979-80 | USA | Psychoactive substance use: tobacco | 22 schools, 697 pupils | 6-8 grades | NR | Quantitative | Cross-sectional design (program effects) and experimental design (intervention) |
| **36** | **Fuertes 2022 [115]** | NR | Spain | Public health: violence | 118 pupils | 10-13 years | 64.4 | Mixed methods | Quasi experimental and interviews |
| **37** | **Ghimire 2020 [124]** | NR | Nepal | SRH | 166 pupils, 10 teachers, 8 School Management Committee, 10 Parents, 2 local leaders | 10-14 years | 54 | Qualitative | Interview/focus group/group interview |
| **38** | **Giles 2001 [28]** | NR | USA | Psychoactive substance use: drugs | 3 schools, 328 pupils, 24 teachers | 6-7 grades | 53.6 | Quantitative | Single arm before and after |
| **39** | **Giles 2010 [29]** | 2004-2005 | USA | Psychoactive substance use: alcohol, tobacco, drugs | 1080 pupils, 48 teachers | 6-7 grades | 51.7 | Quantitative | Single arm before and after |
| **40** | **Gonzales 2004 [26]** | 2000-2001 | USA | Psychoactive substance use: tobacco | 3 schools, 448 pupils | 10th grade | 53.5 | Quantitative | Quasi-experimental |
| **41** | **Hanewinkel 2004 [27]** | 1998-1999 | Austria | Psychoactive substance use: tobacco | 1024 pupils, 55 teachers | 5-6 grades | 49.9 | Quantitative | Quasi-experimental |
| **42** | **Haruna 2018 [94]** | NR | Tanzania | SRH | 1 school, 80 pupils | 11-15 years | 47.5 | Mixed methods | Individually randomized and interview/focus group/group interview |
| **43** | **Hassan 2014 [40]** | NR | Lebanon | Mental health | 80 pupils | 7-9 years | NR | Quantitative | Quasi-experimental |
| **44** | **Hecht 2006 [41]** | 1998-2000 | USA | Psychoactive substance use: drugs | 35 schools, 6298 pupils | 7th grade | 47 | Quantitative | Non-randomized interventional |
| **45** | **Heo 2021 [76]** | 2017-2019 | USA | Nutrition, mental health, physical activity | 21 schools, 1463 pupils | 15-16 years | 55.7 | Quantitative | Quasi-experimental |
| **46** | **Jacque 2016 [42]** | 2010-2013 | USA | Public health | 5 schools, 398 pupils | 11-12 grades | NA | Quantitative | Quasi-experimental |
| **47** | **Johnson 1985 [43]** | 1981- 1983 | USA | Psychoactive substance use: tobacco | 9 schools, 3574 pupils | NA | NA | Quantitative | Longitudinal study |
| **48** | **Jones 2022 [141]** | 2022 | USA | Public health: pandemic | 4 schools | Junior and senior pupils | NR | Qualitative | Action research |
| **49** | **Kafewo 2008 [120]** | 2005 | Nigeria | SRH | 1 school, 15 pupils, 2 teachers | 14-15 years | 100 | Qualitative | Ethnography/participant observation, |
| **50** | **Kapp 1980 [96]** | 1976-1977 | USA | SRH | NR | NR | NR | Mixed methods | Single arm before and after and evaluation forms |
| **51** | **Kärkkäinen 2018 [122]** | 2014 | Finland | Public health | 1 school, 21 pupils | 12-13 years | NR | Qualitative | Interview/focus group/group interview |
| **52** | **Kärkkäinen 2019 [121]** | NA | Finland | Nutrition | 1 school, 43 pupils, 1 teacher, 3 student teachers | 13-14 years | 32.6 | Qualitative | Open-ended questionnaires, content analysis |
| **53** | **Keselman 2007 [109]** | NR | China | SRH: AIDS/HIV | 1 school, 27 pupils | 7th grade | NR | Mixed methods | Quasi-experimental |
| **54** | **King 2008 [66]** | NR | USA | Public health | 3 schools, 1365 pupils | 15-19 years | 49 | Quantitative | Cross-sectional  survey |
| **55** | **Klim-Conforti 2023[80]** | 2018-2019 | Canada | Mental health | 1 school, 196 pupils | 11-14 years | NR | Quantitative | Randomized controlled  trial |
| **56** | **Kocken 2015 [67]** | NR | Netherlands | Nutrition | 12 schools, 303 pupils | 12-14 years | 54.7 | Quantitative | Cluster randomized |
| **57** | **Kostanjevec 2017 [110]** | NR | Slovenia | Nutrition | 1 school, 13 pupils | NR | NR | Mixed methods | Quasi-experimental and interview/individual semi-structured interview |
| **58** | **König 2022 [81]** | 2021-2022 | Germany | Public health | 323 pupils | 16-20 | 58.2 | Quantitative | Cross-sectional surveys |
| **59** | **Kupersmidt 2010 [59]** | NR | USA | Psychoactive substance use: alcohol, tobacco | 12 schools, 344 pupils | 7-13 years | 51 | Quantitative | Cluster randomized |
| **60** | **Lakin 2008 [102]** | 2002-2005 | UK | Nutrition | 2 schools | 7-8, 10-11 years | NR | Qualitative | Interview/focus group/group interview, observations |
| **61** | **Layzer 2017 [103]** | 2012-2014 | USA | SRH | 7 schools, 1527   pupils, 116 peer educators | 9th grade | 52 | Mixed methods | Cross-sectional survey and observations, focus group/group interview |
| **62** | **Lin 2021 [60]** | NR | Taiwan | Psychoactive substance use: drugs | 14 schools, 648 pupils | 13-14 years | 49 | Quantitative | Quasi-experimental |
| **63** | **Manesis 2022 [139]** | 2021-2022 | Greece | Public health: violence | 20 pupils | 6^th^ grade | 45 | Qualitative | Action research |
| **64** | **Mason –Jones 2011 [38]** | 2007-2008 | South Africa | SRH | 30 schools, 3934 pupils | 15-16 years | 57 | Quantitative | Quasi-experimental |
| **65** | **Maticka-Tyndale 2010[100]** | 2001-2004 | Kenya | SRH: AIDS/HIV | 60 schools, 3403 pupils, 40 teachers | 11-17 years | 44-45 | Mixed methods | Quasi-experimental, survey, interview, focus group |
| **66** | **Marqes 2013 [104]** | 2011-2014 | USA | SRH | 10 schools | 14-16 years | NR | Mixed methods | Cluster randomized and interview/ focus group/group interview |
| **67** | **Marshman 2021[140]** | NR | UK | Somatic health | 40 schools, 4000 pupils | 11-13 years | NR | Qualitative | Action research |
| **68** | **Mesman 2021 [82]** | 2017-2019 | Netherlands | Nutrition, Physical activity, Psychoactive substance use: alcohol | 10 schools, 1056 pupils | 16+ | 54.7 | Quantitative | Pre- and post intervention questionnaire |
| **69** | **Midford 2013, 2014 2016 [46-48]** | 2010-2012 | Australia | Psychoactive substance use: alcohol, tobacco, drugs | 21 schools, 1752 pupils | 13-15 years | 64.3 | Quantitative | Cluster randomized |
|  |  |  |  |  |  | 13-16 years | 54.3 |  |  |
|  |  |  |  |  |  | 13-14 years | 54 |  |  |
| **70** | **Modell 2023 [116]** | 2014-2019 | USA | Somatic health, psychoactive substance use: drugs | 2 schools, 1171 | 6^th^ – 8^th^ grade | 49.4 | Mixed methods | Controlled-before and after  , interviews |
| **71** | **Moreno 2018[49]** | NA | USA | SRH: AIDS/HIV | 624 pupils | 12-17 years | NA | Quantitative | Cross-sectional survey |
| **72** | **Moreira 2010 [44]** | 2002-2006 | Portugal | Mental health | 5145 pupils | 6-10 years | 50 | Quantitative | Quasi-experimental |
| **73** | **Neumann 1999 [45]** | 1997-1998 | USA | Public health | 8 schools, 120 pupils | 4-12 grades | 57 | Quantitative | Observational |
| **74** | **Nielsen 2023 [143]** | NR | Nepal and Bangladesh | SRH, mental health | 22600 | 10-19 | NR | NR | NR |
| **75** | **Nygard 2021 [132]** | 2017 | Finland | Nutrition, somatic health | 1 school, 39 pupils, 2 teachers | 8th grade | NR | Qualitative | Interview/individual semi-structured interview |
| **76** | **Nsangi 2017 [106]** | 2016 - NA | Uganda | Public health | 60 schools | 10-12 years | NR | Mixed methods | Cluster randomized |
| **77** | **O'Hara 1996[62]** | 1993-1994 | USA | SRH: AIDS/HIV | 1 school, 77 students | 15-20 years | 45.5 | Quantitative | Cross-sectional survey |
| **78** | **Orsini 2019 [61]** | 2001-2004 | USA | Psychoactive substance use: alcohol, tobacco, drugs | 26 schools, 3467 pupils, 27 teachers | 13-18 years | 52.3 | Quantitative | Cluster randomized |
| **79** | **Pacheco 1991 [131]** | 1988-1989 | USA | SRH: AIDS/HIV, Psychoactive substance use: drugs | 1 school, 648 pupils | 9-12 grades | 50 | Qualitative | Interview/individual semi-structured interview |
| **80** | **Palmer 2018 [39]** | 2011 | USA | Physical activity | 2 schools,  343 pupils  6 teachers | 12-14 years | 50 | Quantitative | Quasi-experimental |
| **81** | **Paul 2019 [97]** | NR | Brunei | Somatic health | 1 school,  21 pupils | 14-15 years | 62 | Mixed methods | Controlled-before and after and interview/focus group/group interview |
| **82** | **Petrie 2017 [98]** | 2015 | Scotland | Psychoactive substance use: alcohol | 1 school,  13 pupils | 15-16 years | NA | Mixed methods | Case study and interview/focus group/group interview |
| **83** | **Perry 1989 [34]** | 1983-1987 | USA | Nutrition | 1100 pupils | 11-16 years | NR | Quantitative | Cross sectional survey; non-randomized interventional |
|  | **Kelder 1995 [33]** |  |  |  | 5 schools, 1342 pupils | 6-12 grades | NR | Quantitative | Non-randomized interventional |
| **84** | **Pieczka 2019 [125]** | 2013-2015 | Scotland | Psychoactive substance use: alcohol | 6 schools, 3000 pupils | 14-18 years | NA | Qualitative | Interview/individual semi-structured interview |
| **85** | **Ponsford 2021 [138]** | 2017 | UK | SRH | 23 schools,  75 pupils.  23 practitioners and policymakers | 13-15 years | NA | Qualitative | Interview/ focus group/group interview |
| **86** | **Porcu 2022 [83]** | 2020-2022 | Italy | Public health | 14 schools, 896 pupils | 7-9 years | NR | Quantitative | Pre- and post intervention questionnaire |
| **87** | **Rajan 2017 [99]** | NA | USA | Somatic health | 2 schools, 168 pupils, 5 teachers | 6th grade | NA | Mixed methods | Quasi-experimental and classroom observation 77data and written details from  the teachers |
| **88** | **Reubsaet 2005 [64]** | NR | Netherlands | Public health | 39 schools, 2868 pupils | 15-18 years | 55 | Quantitative | Randomized controlled trial |
| **89** | **Resnicow 1993 [63]** | 1979 –1985 | USA | Public health, nutrition, physical activity, psychoactive substance use, somatic health, lifestyle | Study I - 37 schools, 2474 pupils | 4-9 grades | NR | Quantitative | Cluster randomized |
|  |  | 1983-1988 |  |  | Study II – 9 schools, 1063 pupils | 4- 6 grades |  |  |  |
|  |  | 1987- 1990 |  |  | Study III – 5 schools, 2973 pupils | 1-4 grades |  |  |  |
| **90** | **Riggs 2007 [65]** | NR | USA | Nutrition | 2 schools, 54 pupils | 5th grade | 58 | Quantitative | Single arm before and after |
| **91** | **Ridge 2002 [133]** | 1997-NR | Australia | Public health, mental health, nutrition | 100 schools | NR | NR | Qualitative | Interview/ focus group/group interview |
| **92** | **Rogow 2013 [108]** | 2009-2012 | Nigeria | SRH | 51 schools, 4337 pupils, 508 teachers | NA | NA | Mixed methods | Observation, interview,  Survey, focus group discussion |
|  |  |  | China |  | 6 schools, 1500 pupils, 30 teachers | 16 years |  |  |  |
| **93** | **Ruge 2016 [134]** | 2012-2013 | Denmark | Nutrition | 1 school, 100 pupils  1 school, 28 pupils  1 school, 36 pupils | 13-14 years | NR | Qualitative | Action research |
| **94** | **Santos-Beneit 2019[73]** | 2014, 2017, 2020 | Spain | Nutrition, physical activity, somatic health, mental health | 24 schools | 6-11 years | NR | Quantitative | Cluster randomized |
| **95** | **Seal 2006 [50]** | NA | Thailand | Psychoactive substance use: tobacco, drugs | 2 schools, 170 pupils | 15 years | 10.6 | Qualitative | Controlled-before and after, survey |
| **96** | **Schonfeld 2001[107]** | 1997-1998 | USA | Somatic health | 1 school, 67 pupils | 5-11 years | NR | Mixed methods | Pre- and post-test, interviews |
| **97** | **Scull 2022 [84]** | 2019-2020 | USA | SRH | 17 schools, 590 pupils | 9-10 grades | 53 | Quantitative | Randomized control trial |
| **98** | **Shah 2011[127]** | NA | Australia | Nutrition, physical activity | 3 classes | 13-14 years | NA | Quantitative | Survey |
| **94** | **Shah 2017 [126]** |  |  |  | 22 schools |  |  |  | Cross-sectional survey, controlled-before and after |
| **99** | **Shensa 2016 [51]** | 2012 | USA | Psychoactive substance use: tobacco | 1 school, 135 pupils | 9th grade | 54 | Quantitative | Randomized crossover design |
| **100** | **Shinde 2017**  **[95]** | 2015-2016 | India | SRH: AIDS/HIV psychoactive substance use, mental health | 49 schools, 16973 pupils, 25 teachers | 13-15 years | 45 | Mixed methods | Cluster randomized  and interview/focus group/group interview |
|  | **Shinde 2020 [32]** |  |  |  |  |  |  | Quantitative | Cluster-randomized; repeat cross-sectional study |
| **101** | **Simões 2021[89]** | 2017-2018 | Portugal | Mental health | 22 schools, 1084 pupils, 123 teachers and school staff | 4-15 years | 46.8 | Quantitative | Cross-sectional survey |
| **102** | **Simon 2022 [85]** | NR | Germany | Nutrition, physical activity, mental health | 76 pupils | 13-14 years | 56.6 | Quantitative | Quasi-experimental |
| **103** | **Timol 2016 [31]** | 2012-2013 | South Africa | SRH | 236 schools, 28320 pupils | 10-17 years | 55 | Quantitative | Cluster randomized |
| **104** | **Tiwari 2020[30]** | NR | India | Public health | 2 schools, 274 pupils | 6-8 grades | NR (majority male) | Quantitative | Quasi-experimental |
| **105** | **Türkyılmaz 2022 [86]** | 2020-2021 | Turkey | Nutrition | 1 school, 45 pupils | 2^nd^ grade | 55.6 | Quantitative | Quasi-experimental |
| **106** | **Vieira R 2016 [101]** | NR | Portugal | Somatic health | 1 school, 22 pupils | 11-12 years | 59 | Mixed method | Action research, cross-sectional survey |
| **107** | **Velasco 2017 [52]** | NR | Italy | Psychoactive substance use: drugs | 55 schools, 3048 pupils | 11 years | 51 | Quantitative | Quasi-experimental |
| **108** | **Venditti 2009 [53]** | 2006-2009 | USA | Lifestyle: nutrition and physical activity | 42 schools, 6573 pupils | 5-8 grades | 52.3 | Quantitative | Cluster randomized |
| **109** | **Wang 2022 [137]** | 2020 | China | Physical activity | 2 schools, 10 pupils | 10-12 years | 50 | Qualitative | Interview |
| **110** | **Werle 2004 [128]** | NR | USA | Public health: violence | 1 school, 13 pupils | 13-15 years | 46 | Qualitative | Grounded theory |
| **111** | **Wiist 1991 [56]** | NR | USA | Psychoactive substance use: tobacco | 7 schools, 347 pupils, 4 teachers | 11-12 years | 48 | Quantitative | Non-randomized interventional |
| **112** | **Williams 2023 [87]** | NR | USA | Public health: violence | 14 schools, 699 pupils | 11-13 years | 52.7 | Quantitative | Cluster randomized |
| **113** | **Wolfe 2009 [54]** | 2004-2007 | Canada | Public health: violence | 20 schools, 1722 pupils | 14-15 years | 52.8 | Quantitative | Cluster randomized |
|  | **Wolfe 2012[55]** |  |  |  | 6 schools, 196 pupils |  | 56 |  | Observation |
| **114** | **Yoon 2021 [88]** | 2018-2019 | Vietnam | Mental health | 70 schools, 2958 pupils | 11-13 years | 51 | Quantitative | Cluster-randomized controlled trial |
| **115** | **Zion 2021 [113]** | NR | NR | Psychoactive substance use: alcohol | 4 schools, 402 pupils | 10-12 years | 49.8 | Mixed methods | Cluster randomized, survey, interview |
| *NR – not reported; NA – not applicable; | | |  |  |  |  |  |  |  |
|  | | | | | | | | | |
