# Supplementary material for: Teaching methods for critical thinking in health education of children up to high school: A scoping review
Source: PLoS One. 2024 Jul 18;19(7):e0307094. doi: 10.1371/journal.pone.0307094 (PMC11257347; doi:10.1371/journal.pone.0307094)
Supplement: Attachment — Submitted filename: Response to Reviewers.docx [file pone.0307094.s004.docx]

Dear Editor,

Thank you for providing us with your remarks and two reviews. We have addressed all the comments and corrected the manuscript accordingly.

We have added the statement about the role of the Founder: "The funders had no role in study design, data collection and analysis, decision to publish, or preparation of the manuscript." Additionally, we have requested adding an Acknowledgment statement as follows: We thank dr Magdalena Koperny for creating the search strategy.

| **Teaching methods for critical thinking in health education of children up to high school: a scoping review** | |
| --- | --- |
| Reviewer 1 |  |
| The scoping review followed JBI and PRISMA, the gold standards, for this review paper. It is beautifully written with no grammatical or editorial mistakes. It is interesting to see that the authors used MASQDA to conduct the qualitative synthesis and reported the results based on the six dimensions. The methodology is sound. The results are clear and well-organized. The discussion is not as strong as Methods and Results but acceptable. I do have a question regarding 1056 studies. When I calculated the numbers listed in the first paragraph of Results, the total was 1053. | Thank you, Reviewer 1, for taking the time to review our work. We appreciate your kind comments and positive feedback.  Thank you for paying our attention to our calculation of the studies sought for retrieval. We have verified it and cleared it out in the text.  Our calculation looks as follows: the total number of the studies sought for retrieval is 1056, of which:   - 938 were excluded due to 10 different reasons - 3 potentially eligible studies were identified as ongoing - 115 eligible studies were included.   We revisited the explanation of this calculations that we provided initially in the manuscript and cleared it out. Now it states:  *We identified 118 eligible studies, of which 3 were still ongoing [43-45]. Finally, we included 115 completed studies.* |
| Reviewer 2 | |
| I want to applaud the authors for their database selection, reporting the dates when searches were last executed, and sharing their entire search strategies in the supplementary materials. For the most part, they are reproducible. To ensure reproducible search methods, the authors will want to indicate which platforms were used for all databases. This was done with some (e.g., Ovid Medline) but not all (e.g., EMBASE via ?).  I have a concerns about the comprehensiveness of the literature search. For some of the databases, there were only subject heading searches performed without targeting other metadata fields like titles, abstracts, and author keywords. I worry that some studies could have been missed in this approach. Could you justify this choice? | Thank you, Reviewer 2, for your positive feedback on our work and for your detailed comments. We appreciate the time and efforts you have put into reviewing our manuscript. Below we respond to your remarks.   1. Thank you for the comment regarding the platforms.   We used the following platforms to search the databases:   - Embase from Elsevier - WoS from Clarivate - Medline from Ovid - ERIC from EBSCO - PsycArticles from EBSCO - CINAHL from EBSCO - Proquest from Proquest central   We added to the missing details to the Supporting information 2.   2. As far as comprehensiveness of the literature search, we have tested several strategies to choose the option that would be optimal and feasible for research question. In the main databases (Medline and Embase) we used a comprehensive approach, and we used MesH Tesaurus terms and fields. In Medline via Ovid .mp which means that database search title, abstract, original title, name of substance word, subject heading word, protocol supplementary concept word, rare disease supplementary concept word, unique identifier). In Embase we used an explode option (major topic), index term and some keywords we searched in all fields ('teaching' OR 'curriculum' OR 'education’/ 'critical thinking' OR 'thinking'). Web of Science Core Collection employs no controlled vocabulary or thesaurus, so we used abstract, title, author keywords. In CINHAL database we used Word in Subject Heading, Exact Subject Heading and for some keywords, such as "critical thinking", we used all text search. For some terms in Proquest Central, like “Health education”, we used the main subject option because when we had used a broader search without restrictions, we have got huge number of results, and when we provide initial selection, most of the records were irrelevant. Additionally, even after narrowing down the search fields in databases like ProQuest and CINHAL, a high number of results persisted and was screened. During the selection process, many records were still excluded.   We believe that searching across multiple databases minimizes the risk of overlooking crucial publications pertinent to the research problem under analysis. |
